# Minimally Invasive Posterior Stabilization Improved Ambulation and Pain Scores in Patients with Plasmacytomas and/or Metastases of the Spine

**DOI:** 10.1155/2011/239230

**Published:** 2011-09-05

**Authors:** Joseph H. Schwab, Alessandro Gasbarrini, Michele Cappuccio, Luca Boriani, Federico De Iure, Simone Colangeli, Stefano Boriani

**Affiliations:** ^1^Department of Orthopedic Surgery, Massachusetts General Hospital, Yawkey 3, 55 Fruit Street, Boston, MA 02114, USA; ^2^Department of Oncologic Surgery, Rizzoli Institute, 40136 Bologna, Italy; ^3^Department of Orthopedic Surgery, Ospedale Maggiore, 40136 Bologna, Italy; ^4^Department of Orthopaedics, Traumatology and Spine Surgery, Ospedale Maggiore AUSL, 40136 Bologna, Italy

## Abstract

*Background*. The incidence of spine metastasis is expected to increase as the population ages, and so is the number of palliative spinal procedures. Minimally invasive procedures are attractive options in that they offer the theoretical advantage of less morbidity. *Purpose*. The purpose of our study was to evaluate whether minimally invasive posterior spinal instrumentation provided significant pain relief and improved function. *Study Design*. We compared pre- and postoperative pain scores as well as ambulatory status in a population of patients suffering from oncologic conditions in the spine. *Patient Sample*. A consecutive series of patients with spine tumors treated minimally invasively with stabilization were reviewed. *Outcome Measures*. Visual analog pain scale as well as pre- and postoperative ambulatory status were used as outcome measures. *Methods*. Twenty-four patients who underwent minimally invasive posterior spinal instrumentation for metastasis were retrospectively reviewed. *Results*. Seven (29%) patients were unable to ambulate secondary to pain and instability prior to surgery. All patients were ambulating within 2 to 3 days after having surgery (*P* = 0.01). The mean visual analog scale value for the preoperative patients was 2.8, and the mean postoperative value was 1.0 (*P* = 0.001). *Conclusion*. Minimally invasive posterior spinal instrumentation significantly improved pain and ambulatory status in this series.

## 1. Introduction

It is estimated that over 1.5 million new cases of cancer occur each year in the United States. Roughly 500,000 people die each year in the United States from cancer-related causes, most of whom have metastatic disease [1]. The three most common cancers, lung, breast, and prostate, all commonly spread to bone, and the spine is the most frequently involved segment of the skeleton [2]. The majority of spinal metastases are asymptomatic and do not require local treatment. Radiation is the standard of care for painful spinal metastases in the absence of an unstable fracture or impending fracture [3]. In cases where a fracture is unstable or there is an impending fracture, then stabilization ought to be considered. 

Minimally invasive techniques offer potential advantages over open techniques particularly in the population of patients suffering from metastatic disease of bone. Minimally invasive techniques, as the name implies, are potentially associated with less soft tissue injury than their open surgical counterparts [4]. Furthermore, minimally invasive posterior stabilization has been shown to be associated with relatively low blood loss [5]. This may translate to less morbidity and possibly shorter hospital stays. In addition, the use of minimally invasive techniques may be associated with lower postoperative infections. Posterior stabilization allows for immediate mobilization without the need for external bracing. This is particularly important in this patient population as the main goal ought to be to maintain their quality of life. 

We present the short-term followup of 24 cases of metastatic disease in the spine treated minimally invasively with posterior instrumentation. We compared preoperative ambulatory status and preoperative pain levels with postoperative levels. Our goal was to determine whether minimally invasive posterior instrumentation could provide meaningful improvement in pain control and ambulatory status in the short term.

## 2. Materials and Methods

We performed a retrospective review of 24 consecutive cases treated with minimally invasive posterior spinal instrumentation for spine tumors. The patients were followed for an average of 9 months (range 3–21). There were 13 male and 11 female patients. The average age was 62 (range 33–86). Patients with spinal cord compression are not suitable candidates for this procedure and thus were excluded. The following cancer diagnoses were included: plasmacytoma (9), metastatic breast cancer (5), metastatic hepatocellular carcinoma (2), metastatic lung cancer (1), metastatic prostate cancer (1), metastatic colon cancer (2), metastatic angiosarcoma (1), metastatic liver cancer (1), metastatic thyroid cancer (1), and lymphoma (1). The thoracic spine was the primary site of disease in 10 cases, and the lumbar spine was the primary site of disease in 14 cases. 

Only cases where instrumentation was used were included in this paper. The indications for instrumentation were made on a case-by-case basis. In general, an unstable fracture or an impending fracture were indicated for surgery. The decision to proceed with surgery was made on a case-by-case basis. Patients with mechanical back pain, defined as pain with movement which is relieved by rest, that corresponded to an area of metastases were considered for surgery. 

We were interested in examining whether surgical stabilization had a statistically significant impact on ambulatory status and self-reported pain levels. We categorized patients into one of two categories with regard to ambulation. If they were able to ambulate with or without a gait aid, they were given a score of 1. If they were unable to ambulate, then they were given a score of 0. We utilized the Fisher's exact test to evaluate whether the patients' ambulatory status was improved by our intervention. We used a visual analog scale (VAS) to assess pain as reported by the patients. A score of 1 was given for mild or no pain (0–3). A score of 2 was given for moderate pain (4–6), and a score of 3 was given for severe pain (7–10). The student's *t*-test was used to compare the mean VAS between preoperative and postoperative groups. A *P* value of less than 0.05 was used to determine whether a value was statistically significant.

The procedure involves the use of fluoroscopic imaging in order to place the pedicle screws percutaneously. Adequate imaging is required to visualize the pedicles well in both the lateral as well as a/p views. This may require the radiology technician to cant the fluoroscope in order to visualize the pedicles clearly. This is particularly true in the sacrum. A radiopaque marker is placed on the skin so that the incisions are appropriately placed just lateral to the pedicles. This allows medialization of the trochar. The trochar is passed through the soft tissues down to the bony surface. Prior to penetrating the cortex with the trochar, it is important to confirm that the trochar is on the lateral border of the pedicle silhouette. In addition, it is useful to place the trochar along the superior quarter of the pedicle as seen on the a/p and lateral image. Placement of the trochar in this manner allows one to medialize the pedicle screw as it passes into the vertebral body. After the trochar has been successfully placed into the vertebral body, a guide wire is placed through it. The trochar is then removed, and a series of dilators are passed over the wire. Each system has a slightly different mechanism of screw/rod placement, and thus the technique should be tailored to the implant used as well as particular anatomy of the patient. The unifying theme behind these systems is that they provide a percutaneous/minimally invasive means by which they can stabilize the spine.

## 3. Results

Seven (29%) of the 24 patients were unable to ambulate secondary to pain and instability prior to surgery (Table 1). All 24 patients were ambulating within 2 to 3 days after having surgery (*P* = 0.01). The mean visual analog scale value for the preoperative patients was 2.8, and the mean postoperative value was 1.0 (*P* = 0.001).

Twenty-one of 24 patients presented with severe pain. Seven patients were unable to ambulate secondary to pain. One patient complained of radicular pain in addition to their back pain. The rest of the patients complained primarily of back pain. 

The two patients who presented with minimal back pain had lytic lesions that were concerning for impending collapse. Both of them were to undergo surgery for pathologic fractures of their limbs. In one case a proximal femoral replacement was performed secondary to a pathologic fracture. The other patient underwent a proximal humerus resection secondary to pathologic fracture. Both of these patients underwent minimally invasive spinal stabilization under the same anesthetic. Both of these patients were in need of chemotherapy. It was felt that their spines were going to collapse secondary to the lytic nature of their lesions. It was determined that minimally invasive stabilization under the same anesthetic as the one used for their limb reconstructions made the most sense. If their spines became a problem in the ensuing months, then their chemotherapy would have to be interrupted in order to stabilize their spines. This decision was made in conjunction with the patients and their medical oncologists.

Another patient with metastatic hepatocellular carcinoma underwent open decompression and stabilization for high-grade spinal cord compression in the thoracic spine. They had a painful lumbar metastasis at L4 and L5, which was stabilized minimally invasively from L3-S1 under the same anesthetic.

**Figure 1:**
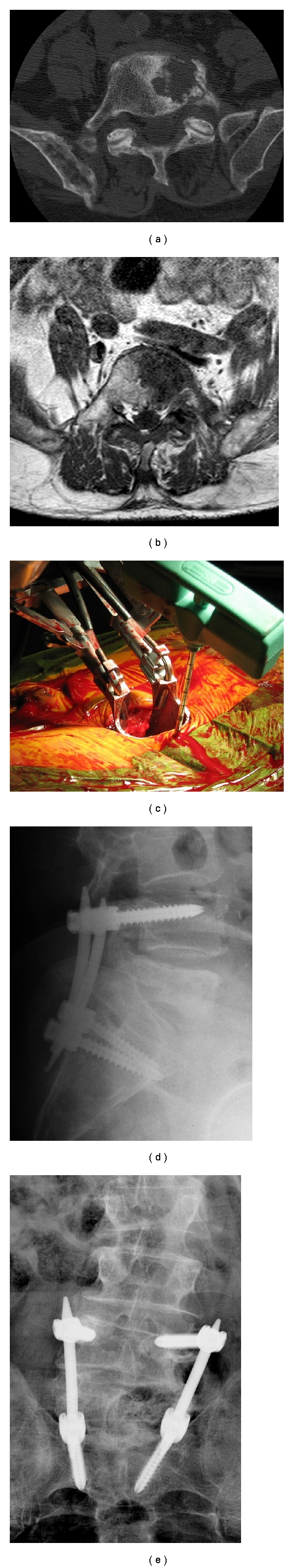
(a) This is a preoperative axial CT image of the L5 vertebrae demonstrating a lytic lesion from metastatic prostate cancer, (b) this preoperative axial MRI image demonstrates compression of the L5 nerve root on the left side, (c) this intraoperative photo demonstrates a trochar utilized to localize the pedicle prior to pedicle screw insertion, and it also demonstrates the minimally invasive access utilized for decompression of the L5 nerve root, (d) and (e) these are the postoperative a/p and lateral images demonstrating the L4-S1 instrumentation.

One 86-year-old patient with metastatic prostate cancer presented with back pain and radicular pain in an L5 distribution. He underwent a minimally invasive decompression along with minimally invasive stabilization (Figure 1).

Three patients had deformities associated with pathologic fractures. In two instances the patients had kyphotic deformities, and in the other case the patient had scoliosis. All three deformities were noted in the lumbar spine. The kyphotic deformities measured 25° and 15° over the involved lumbar vertebrae. The scoliosis measured 15° around L1. All three of these patients were managed with minimally invasive posterior instrumentation. The kyphotic deformities improved by 10° and 9°, respectively, and the scoliosis improved by 13°.

## 4. Discussion

We report the successful management of 24 patients treated with minimally invasive posterior spinal instrumentation for malignancies of the spine. The patients had a statistically significant improvement in ambulatory status as well as pain levels after their minimally invasive stabilization. All patients in our series were ambulatory without a brace after surgery. The rationale behind this form of treatment is that it balances the need to stabilize the spine while avoiding the morbidity associated with open procedures [4].

It is important to maintain proper oncologic perspective when managing this patient population. Many of these patients do not have long to live, and the goal must be to improve or maintain their quality of life during the remaining time. This concept is predicated on the notion that appropriate staging and diagnostic work-ups have been performed prior to rendering treatment. Anecdotally, we have had several patients sent to us for management of their metastatic disease when in fact they had spondylodiscitis. These patients had a history of cancer, and it was assumed that their spine pathology was related. The opposite situation has also occurred in which a patient was thought to have an infection when in fact they had metastatic disease. A biopsy should be performed and cultures should be sent before deciding on and rendering treatment.

While there seems to be an important role for minimally invasive procedures in this patient population, there are instances in which minimally invasive approaches are not appropriate. In the setting of high-grade spinal cord compression, percutaneous procedures should not be entertained. At this time, percutaneous fusions are unproven and if a patient requires a fusion, they should not be treated percutaneously. Furthermore, minimally invasive instrumentation is to be used alongside other adjuvant therapies such as radiation or chemotherapy. If a tumor is not sensitive to either, then one should pause before using a minimally invasive approach.

Traumatic fractures of the thoracolumbar spine have been treated successfully using a similar approach [5, 6]. It is important to note that this procedure does not involve a fusion. The instrumentation should be considered as an internal brace. In theory the instrumentation would eventually fail, and thus it should be removed prior to this occurring, which is often done in the case of traumatic fractures. However, this is meant as a definitive procedure in the setting of metastatic disease. Surgery is meant as a means by which the quality of life of the patient can be improved, and it is not meant as curative. In this way, percutaneous instrumentation is sound from an oncologic perspective. 

Recent studies have questioned the utility of percutaneous cement augmentation of osteoporotic vertebral fractures [7–9]. Currently, it is an accepted means to treat many symptomatic vertebral metastases [10]. However, further studies are needed to prove its utility in patients with spine metastases. Furthermore, the rate of polymethylmethacrylate (PMMA) leakage is between 10 and 40%, and it has been reported to be much higher when CT is routinely used following procedure [11–14]. While most cases of cement leakage are reported to be asymptomatic, there are reports of cement leakage that required urgent surgical decompression [15–18]. In addition, there are reports of symptomatic pulmonary emboli after cement augmentation [19–22]. Further, the use of PMMA is a relative contraindication when the posterior cortex of the vertebral body is breeched by tumor. There have been no trials comparing the use of PMMA augmentation with that of percutaneous fixation. 

Close consultation with medical and radiation oncology is an important component care in these cases. Survival expectations must be discussed and the treatment rendered tailored to each individual case. Local radiation is often an important adjuvant in the setting of spinal metastases, and this is particularly true when one is considering a minimally invasive approach. The goals of surgery are to stabilize and/or decompress the spine. Debulking of tumor is possible in a minimally invasive fashion, but if tumor debulking is a central part of the local control plan, then an open procedure may be more suitable. Local failure due to tumor regrowth is a concern in the setting of minimally invasive approaches, and one is relying more heavily on radiation/chemotherapy when approaching these cases in a minimally invasive fashion. Our study demonstrates the short-term successes associated with minimally invasive approaches to spine metastasis. However, longer followup is needed to assess whether local failure, whether from tumor regrowth or hardware failure, becomes a problem. 

 We report the successful short-term treatment of 24 patients with a minimally invasive approach for malignancies in the spine. Pain and ambulatory status were both improved after this minimally invasive approach. The role of minimal access surgery continues to evolve, and further studies are needed to elucidate the most appropriate patients for this approach. 

## Figures and Tables

**Table 1:** Minimally invasive posterior stabilization for malignancies in the spine.

Sex	Age	Diagnosis	Walking pre-op.	Walking post-op.	Pre-op. pain	Post-op. pain	Pathology level	Instr. levels	Δ Deformity	Time (min)
M	68	Plasmacy.	Y	Y	2	1	L3	L2-4		110
M	86	Metastatic prostate ca.	N	Y	3	1	L5	L4-S1	9° Kyphosis	180
F	65	Plasmacy.	Y	Y	1	1	T10	T9–T11		60
M	80	Metastatic colon ca.	N	Y	3	1	L3-L4-L5	L2-S1		80
F	44	Metastatic breast ca.	Y	Y	3	1	T7	T5–T9	10° Kyphosis	135
F	58	Metastatic breast ca.	N	Y	2	1	L5	L4-S1		80
F	55	Plasmacy.	Y	Y	3	1	L2	T12, L1–L3		105
F	66	Metastatic angiosarc.	Y	Y	3	1	T11	T9, T10–T12, L1		180
M	61	Metastatic lung ca.	N	Y	1	1	T5	T3–T7		105
M	48	Metastatic HCC	Y	Y	3	1	L4-L5	L3-S1		75
M	75	Plasmacy.	Y	Y	3	1	T10	T9–T11		60
M	33	Lymphoma	Y	Y	3	1	L1	T12-L2	13° Scoliosis	120
M	75	Metastatic HCC	Y	Y	3	1	T11	T10–T12		120
F	60	Metastatic breast ca.	N	Y	3	1	L1	T12-L2		60
F	68	Metastatic colon	Y	Y	3	1	L4	L3–L5		120
M	75	Metastatic liver	Y	Y	3	2	L1	T12-L2		180
M	64	Plasmacy.	Y	Y	3	1	L5	L4-S1		180
F	73	Metastatic breast	Y	Y	3	1	L3	L2–L4		120
M	37	Plasmacy.	N	Y	4	1	T7	T6–T8		120
F	72	Plasmacy.	Y	Y	3	1	T10	T9–T11		180
F	52	Plasmacy.	Y	Y	3	1	L5	L4-S1		180
F	75	Metastatic breast	Y	Y	3	1	T10-T11	T9–T12		180
M	45	Plasmacy.	N	Y	3	1	T10	T9–T11		120
M	59	Metastatic thyroid	Y	Y	4	1	T6-L4	T3-S1		180

Plasmacy.: plasmacytoma, angiosarc.: angiosarcoma, HCC: hepatocellular carcinoma, ca.: cancer, Δ deformity: the measured change in deformity from preoperative to postoperative images, pre and postoperative pain scale 3: severe, 2: moderate, 1: none to mild.
